# Obesity and prevalence of chronic diseases in the 1999–2000 Italian National Health Survey

**DOI:** 10.1186/1471-2458-8-140

**Published:** 2008-04-28

**Authors:** Stefano Calza, Adriano Decarli, Monica Ferraroni

**Affiliations:** 1 Section of Medical Statistics and Biometry, Department of Biotechnologies and Biomedical Sciences, University of Brescia, Italy; 2 Institute of Medical Statistics and Biometry "Giulio A. Maccacaro", University of Milan, Italy, and Statistics and Biometry Unit, Istituto Nazionale per lo Studio e la Cura dei Tumori, Milan, Italy; 3 Department of Medicine, Surgery and Dentistry, Unit of Medical Statistics, University of Milan, Italy

## Abstract

**Background:**

There is consistent evidence that obesity is a correlate of mortality. Less information is available about the relation between body weight and the prevalence of diseases. We investigated the prevalence of overweight and obesity and their relationship with 14 groups of chronic diseases in a Mediterranean population using data from the Italian National Survey collected in 1999–2000.

**Methods:**

A sample of 52,300 families was randomly selected using a complex stratified multistage design, within strata of geographical areas, municipalities, and household sizes, to produce estimates representative of the whole Italian population. Data were collected by civil servants both with an interview and a self-reported questionnaire.

**Results:**

The present study documents an increase in the prevalence of overweight among Italian adults in the last decades and an increased prevalence of several chronic conditions in obese or overweight individuals. A general pattern of a positive association between excess weight and chronic disease was observed for both sexes. The ratio of the prevalences of cardiovascular diseases, diabetes and chronic respiratory diseases was higher in obese versus normal-weight individuals in the age group under 45 years.

**Conclusion:**

To reduce the prevalence of chronic diseases a policy promoting a healthier individual lifestyle is becoming more and more desirable.

## Background

Overweight and obesity have become a well recognized problem in many Western countries since the 1950s [1], mostly as a consequence of improved living conditions. Plentiful availability of food and less physically demanding jobs typical of industrialized and globalized societies are deemed to be the major determinants of the "obesity epidemic". In many Western countries the prevalence of obesity has been increasing steadily. In the United States it rose from 12% in the early 1990s to more than 17% in 1998, with the highest increase in the youngest age groups [2]. Europe as well is experiencing a marked increase in obesity rates, which have doubled over just a few years [3].

The burden of obesity is generally considered to be a correlate of mortality [4-6] and to lead to an increase in the prevalence of chronic diseases [7,8] such as type 2 diabetes mellitus, hypertension, cardiovascular disease, and some of the most relevant cancer types [9,10]. The impact of these diseases in terms of morbidity and mortality, health-care expenditure and individual health-related quality of life is far from negligible [3].

To draw a cross-sectional picture of the recent status of the Italian population, we investigated the prevalence of various chronic diseases in relation to body weight using data from a national survey conducted by the Italian Institute of Statistics in 1999–2000.

## Methods

### Data

In 1999–2000, the Italian Institute of Statistics (hereafter ISTAT) began to collect new multipurpose information on Italian families regarding "Health status and use of health services" [11]. A sample of 52,300 families (for a total of about 140,000 individuals) was randomly selected with a complex stratified multistage design within strata of geographic areas (North, Center, South and Islands), municipalities, and household sizes, to produce estimates representative of the whole Italian population [12]. The households were selected by systematic sampling. When a household had to be replaced, the next on the list of households included in the same stratum was recruited. Replacement was necessary because the sampling size for each stratum was defined by protocol according to [13]. This method allows optimal allocation of the sample size when the aim is to estimate various relevant items with a relative error less than a predefined value (i.e., less than 9% for estimated prevalences greater than 5% in the population). The overall participation rate was satisfactory (less than 10% of the original sample had to be replaced). Interviews were arranged and conducted by civil servants (appointed by each municipality included in the study) at the families' homes and involved all family members. Proxy interviews were allowed for those family members who were absent at the time of the interview. We obtained a copy of the original files regarding the yearly ISTAT surveys relative to the years 1999–2000, with individual information on each interviewed subject.

Data were collected on sociodemographic characteristics including education, employment, daily travel, leisure activities, lifestyle and health conditions, health service utilization, pregnancies, self-reported height and weight, diet, smoking habits, drug consumption and presence of 13 groups of chronic diseases with diagnosis certified by a medical doctor. The disease groups included diabetes mellitus, hypertension, myocardial infarction, angina pectoris, thrombosis (including embolism and cerebral hemorrhage), varices (including varicose veins and varicocele), respiratory diseases (including chronic bronchitis, emphysema and respiratory failure), bronchial asthma, thyroid diseases, osteoarthritis, lower back pain and sciatica, osteoporosis, and cancer (including leukemia and lymphomas).

Myocardial infarction, angina pectoris and thrombosis were grouped together into an additional group including subjects with at least one of these diseases.

Subjects below age 18 were excluded from the analysis, giving a total of 55,303 men and 59,716 women.

### Statistical analysis

The body mass index (BMI) was computed according to the Quetelet formula (weight [kg]/height^2 ^[m2]) and categorized into 5 levels [14,15], namely Underweight (<18.5), Normal (18.5–24.9), Overweight (25–29.9), Obese I (30–34.9) and Obese II/III (≥35). Given the very low prevalence in both sexes of subjects with BMI ≥40 (0.11% of men and 0.17% of women), we collapsed the original obese class II (35–39.9) and class III (≥40) into one category. Standardized morbidity prevalence ratios were computed through direct standardization using the normal weight class as reference.

Odds ratios (ORs) for the prevalence of the selected chronic diseases and their 95% confidence intervals (95%CI) were computed by means of multivariate logistic models [16]. Given the complex sampling design, the Taylor series linearization method was adopted to obtain correct estimates of variance [17-19].

All the logistic regression models used normal BMI class as reference and were adjusted for age, smoking habits and education. Age was fitted as a continuous variable, while smoking was categorized as Never (reference category), Ex-smoker, <15 cigarettes/day, and ≥ 15 cigarettes/day. Education levels were entered as University (reference category), High school, Middle school, and Primary or less.

Tests for trend for the BMI classes were based on a Wald test for the significance of the BMI expressed as a linear term. As the association between health conditions and underweight may depend on different factors, we did not consider the underweight group when testing the BMI linear effect. Sample weights were used in all the cross-tabulations and analyses in order to produce estimates representative at the national level. All analyses were done using the R software [20] and the *survey *package [19].

## Results

### Obesity and sociodemographic factors

According to the ISTAT National Survey, women were slightly older than men (48.9 versus 46.3 years) while BMI was slightly lower in women than in men (men: average 25.3, range 15.1–55.2, women: average 23.7, range 15.1–55.2).

More differences between the sexes became apparent when BMI classification was considered, with 48.9% of men and 32.7% of women being overweight or obese. More specifically, 40.6% of men and 24.6% of women in the ISTAT sample were overweight, while 1.1% of men and 6.3% of women were considered underweight (Table 1). The proportion of obese I subjects was slightly higher in men (7.3% versus 6.6% in women) while the obese II/III prevalence was higher among women (1.5% versus 1.0% in men). Stratifying by age showed that underweight women were mostly young, with a much lower but still sensible percentage in the oldest age class. Men were clearly less frequently underweight in all age groups. By contrast, the proportion of overweight men in all age groups was markedly higher than that of overweight women, while the proportion of obese subjects was roughly the same and increased with age.

**Table 1:** Percent distribution of sociodemographic factors according to BMI in 55,303 men and 59,716 women.

	**BMI (*kg*/*m*^2^)**									
	**Males**					**Females**				
		
	Underweight	Normal	Overweight	Obese I	Obese II/III	Underweight	Normal	Overweight	Obese I	Obese II/III

**Age (years)**										
18–34	2.20	69.36	25.39	2.70	0.35	13.70	74.36	9.68	1.79	0.46
35–44	0.39	48.17	43.41	6.94	1.08	4.88	71.24	18.96	4.01	0.92
45–54	0.37	38.40	49.43	10.19	1.61	2.53	57.74	28.80	8.67	2.26
55–64	0.43	35.91	50.23	11.82	1.61	2.35	48.28	36.33	10.30	2.74
65–74	0.64	37.34	50.21	10.66	1.15	2.16	46.09	37.88	11.64	2.23
≥75	2.44	47.01	43.28	6.56	0.71	5.87	49.40	33.81	9.20	1.72
**Education**										
University	0.90	58.18	35.83	4.62	0.47	8.04	77.34	12.33	1.82	0.47
High school	1.44	58.17	34.74	4.99	0.66	10.37	73.24	13.68	2.18	0.52
Middle school	0.99	50.48	40.62	6.78	1.13	6.65	66.86	20.34	5.05	1.09
Primary or less	1.03	38.25	48.19	11.20	1.34	3.11	45.05	37.44	11.67	2.73
**Geographical area**										
Northwest	1.32	53.25	38.27	6.25	0.90	8.07	63.57	21.29	5.52	1.56
Northeast	0.86	49.28	40.95	7.93	0.98	6.39	61.40	24.32	6.46	1.43
Center	1.03	52.31	39.27	6.55	0.83	5.80	63.53	23.58	5.80	1.28
South	1.06	46.01	43.52	8.14	1.27	4.55	56.23	28.87	8.52	1.82
Islands	1.34	47.38	42.07	8.18	1.03	6.78	59.40	25.56	6.86	1.40
**Smoking habits**										
Never	1.38	53.94	37.74	6.06	0.89	6.16	58.59	26.31	7.25	1.69
Ex-smoker	0.74	38.74	49.08	10.12	1.32	5.09	63.25	24.02	6.07	1.57
< 15 cigarettes/day	1.45	59.88	32.88	5.01	0.78	8.47	69.17	17.44	3.97	0.95
≥ 15 cigarettes/day	0.98	51.40	39.67	7.07	0.88	7.12	66.58	20.10	5.44	0.77
**All**										
%	1.12	49.97	40.62	7.28	1.00	6.34	60.98	24.56	6.60	1.52
N*	252,462	11,270,228	9,162,655	1,643,067	226,158	1,546,994	14,868,975	5,987,887	1,610,073	371,340

Data from the Italian National Health Survey, ISTAT, 1999–2000.

*Numbers are weighted to account for sampling design

Table 1 also presents the distribution of BMI in relation to several sociodemographic factors including education, geographical area, and smoking habits. The proportion of overweight or obese individuals was inversely related to the level of education in both genders. The distribution of underweight subjects was approximately constant in men, while the underweight proportion increased with education level in women.

No particular pattern was apparent in the BMI distribution over geographical areas except for a marked increase in the proportion of overweight and obese women in the South and a decrease in the percentage of underweight subjects in the South, especially if compared to the Northwest. Moreover, the prevalence of overweight and obesity was higher in ex-smoker males.

### Obesity and prevalence of chronic diseases

The morbidity prevalence for various groups of chronic diseases and the standardized morbidity prevalence ratios according to BMI categories and gender are reported in Table 2. For the same diseases, OR estimates adjusted for age, level of education and smoking habits with the corresponding 95% CIs in different categories of BMI are shown for men (Table 3) and women (Table 4).

**Table 2:** Morbidity prevalence (%) and standardized morbidity prevalence ratio (SMPR)* for selected diseases according to BMI class and gender

	**BMI (*kg*/*m*^2^)**					
	
	Overall	Underweight	Normal	Overweight	Obese I	Obese II/III
**Males (n = 55,303)**						
Diabetes mellitus	4.27	1.74 (106)	2.93 (100)	4.85 (144)	9.33 (168)	14.01 (155)
Hypertension	12.44	5.06 (107)	7.82 (100)	15.90 (141)	23.57 (159)	29.66 (141)
Varices	4.15	2.27 (89)	3.41 (100)	4.67 (128)	6.05 (129)	8.40 (109)
Respiratory diseases	5.75	8.45 (103)	4.66 (100)	6.32 (135)	8.84 (153)	11.75 (137)
Bronchial asthma	3.25	4.89 (123)	2.92 (100)	3.26 (115)	4.65 (138)	7.68 (122)
Thyroid diseases	1.01	0.83 (87)	0.74 (100)	1.18 (128)	1.76 (132)	1.46 (132)
Osteoarthritis	16.41	14.83 (112)	12.10 (100)	19.83 (140)	25.64 (156)	27.25 (124)
Lower back pain and sciatica	8.20	5.66 (114)	6.53 (100)	9.71 (124)	11.06 (133)	12.09 (157)
Osteoporosis	1.22	2.39 (141)	1.09 (100)	1.26 (145)	1.55 (130)	2.31 (198)
Cancer	0.97	3.29 (92)	0.85 (100)	0.98 (145)	1.36 (177)	1.44 (119)
Myocardial infarction	1.87	1.82 (99)	1.17 (100)	2.30 (148)	3.57 (172)	7.30 (191)
Angina pectoris	1.05	0.52 (52)	0.71 (100)	1.37 (146)	1.47 (169)	2.18 (169)
Thrombosis	1.05	1.69 (165)	0.79 (100)	1.14 (142)	1.93 (165)	3.19 (127)
MI – Angina pectoris – Thrombosis^1^	3.41	3.56 (112)	2.27 (100)	4.16 (146)	6.05 (170)	10.62 (163)
**Females (n = 59,716)**						
Diabetes mellitus	4.76	0.92 (73)	2.78 (100)	7.57 (173)	13.05 (171)	19.06 (171)
Hypertension	16.32	6.68 (73)	10.60 (100)	26.27 (161)	36.39 (169)	37.98 (151)
Varices	12.32	5.19 (72)	9.37 (100)	17.68 (135)	23.21 (149)	26.68 (141)
Respiratory diseases	4.60	3.12 (86)	3.40 (100)	6.33 (153)	9.35 (142)	10.39 (144)
Bronchial asthma	3.31	2.64 (93)	2.69 (100)	4.23 (129)	5.60 (119)	6.10 (124)
Thyroid diseases	5.47	3.27 (72)	4.76 (100)	6.77 (117)	8.40 (115)	8.91 (105)
Osteoarthritis	27.91	13.40 (70)	21.77 (100)	40.24 (153)	47.59 (160)	50.06 (162)
Lower back pain and sciatica	10.71	4.51 (77)	8.88 (100)	14.60 (127)	16.86 (131)	20.25 (127)
Osteoporosis	9.93	5.81 (69)	7.90 (100)	14.10 (172)	15.82 (187)	15.64 (173)
Cancer	1.29	0.82 (63)	1.06 (100)	1.72 (156)	1.90 (170)	2.62 (177)
Myocardial infarction	0.91	0.63 (77)	0.67 (100)	1.32 (175)	1.79 (181)	1.62 (162)
Angina pectoris	1.12	0.79 (79)	0.79 (100)	1.63 (173)	2.19 (184)	2.76 (166)
Thrombosis	1.06	0.72 (97)	0.76 (100)	1.50 (168)	1.93 (188)	3.83 (129)
MI – Angina pectoris – Thrombosis^1^	2.73	1.87 (82)	1.98 (100)	3.90 (171)	5.12 (186)	7.40 (145)

Data from the Italian National Health Survey, ISTAT, 1999–2000.

*Direct Standardization using the BMI normal class as reference

^1 ^At least one of the indicated diseases

MI, myocardial infarction

**Table 3:** Odds ratio estimates* (95% confidence interval) for the prevalence of chronic diseases in different BMI categories in Italian males

	**BMI (kg/m**^2^**)**									
		
	Underweight		Normal	Overweight		Obese I		Obese II/III		
						
	OR	95% CI†	1‡ (OR)	OR	95% CI	OR	95% CI	OR	95% CI	** *p *for trend
Diabetes mellitus	0.43	(0.19–0.96)	1	1.24	(1.09–1.42)	2.34	(1.95–2.81)	4.21	(2.94–6.03)	<0.01
Hypertension	0.44	(0.27–0.73)	1	1.70	(1.57–1.85)	2.62	(2.31–2.97)	4.14	(3.14–5.46)	<0.01
Varices	0.62	(0.29–1.32)	1	1.10	(0.97–1.25)	1.31	(1.07–1.59)	2.02	(1.31–3.10)	<0.01
Respiratory diseases	1.55	(1.01–2.37)	1	1.00	(0.89–1.11)	1.30	(1.09–1.53)	2.11	(1.49–2.98)	<0.01
Bronchial asthma	1.58	(1.00–2.52)	1	0.87	(0.76–0.99)	1.08	(0.87–1.35)	2.13	(1.41–3.21)	0.37
Thyroid diseases	1.08	(0.53–2.23)	1	1.32	(1.04–1.68)	1.81	(1.25–2.63)	1.43	(0.67–3.05)	<0.01
Osteoarthritis	1.11	(0.81–1.53)	1	1.29	(1.20–1.39)	1.59	(1.42–1.78)	2.00	(1.55–2.58)	<0.01
Lower back pain and sciatica	0.86	(0.56–1.33)	1	1.26	(1.15–1.38)	1.38	(1.20–1.60)	1.61	(1.18–2.19)	<0.01
Osteoporosis	1.49	(0.76–2.93)	1	0.86	(0.68–1.09)	0.99	(0.69–1.42)	1.75	(0.81–3.80)	0.93
Cancer	3.20	(1.56–6.56)	1	0.85	(0.65–1.11)	1.14	(0.72–1.81)	1.36	(0.57–3.27)	0.90
Myocardial infarction	1.21	(0.53–2.75)	1	1.47	(1.21–1.79)	2.13	(1.59–2.87)	5.44	(3.24–9.12)	<0.01
Angina pectoris	0.49	(0.12–1.98)	1	1.39	(1.08–1.78)	1.44	(1.00–2.08)	2.43	(1.15–5.16)	<0.01
Thrombosis	1.35	(0.63–2.90)	1	1.05	(0.82–1.35)	1.71	(1.19–2.46)	3.51	(1.56–7.90)	<0.01
MI – angina pectoris – thrombosis1	1.13	(0.61–2.10)	1	1.37	(1.18–1.58)	1.90	(1.53–2.37)	4.39	(2.83–6.81)	<0.01

Data from the Italian National Health Survey, ISTAT, 1999–2000.

*Adjusted for age, education level and smoking habits

‡ Reference category

**p for trend was evaluated excluding the underweight group

^1 ^At least one of the indicated diseases

MI, myocardial infarction

**Table 4:** Odds ratio estimates* (95% confidence interval) for the prevalence of chronic diseases in different BMI categories in Italian females

	**BMI (kg/m**^2^**)**									
		
	Underweight		Normal	Overweight		Obese I		Obese II/III		
						
	OR	95% CI	1‡	OR	95% CI	OR	95% CI	OR	95% CI	** *p *for trend
Diabetes mellitus	0.37	(0.24–0.58)	1	1.71	(1.51–1.93)	2.96	(2.51–3.48)	5.18	(3.95–6.80)	<0.01
Hypertension	0.72	(0.59–0.88)	1	1.87	(1.74–2.01)	2.92	(2.62–3.26)	3.45	(2.77–4.31)	<0.01
Varices	0.64	(0.52–0.78)	1	1.47	(1.37–1.59)	1.97	(1.76–2.21)	2.48	(2.01–3.07)	<0.01
Respiratory diseases	1.13	(0.86–1.46)	1	1.26	(1.12–1.42)	1.84	(1.54–2.18)	2.25	(1.67–3.03)	<0.01
Bronchial asthma	1.15	(0.85–1.54)	1	1.24	(1.07–1.42)	1.59	(1.30–1.94)	1.79	(1.27–2.54)	<0.01
Thyroid diseases	0.76	(0.59–0.98)	1	1.24	(1.11–1.38)	1.53	(1.29–1.81)	1.67	(1.23–2.28)	<0.01
Osteoarthritis	0.69	(0.59–0.80)	1	1.38	(1.30–1.48)	1.75	(1.58–1.94)	2.16	(1.78–2.62)	<0.01
Lower back pain and sciatica	0.57	(0.46–0.71)	1	1.37	(1.26–1.49)	1.57	(1.38–1.78)	2.06	(1.63–2.59)	<0.01
Osteoporosis	0.84	(0.68–1.06)	1	1.12	(1.02–1.23)	1.20	(1.04–1.37)	1.30	(0.98–1.72)	<0.01
Cancer	0.95	(0.60–1.50)	1	1.14	(0.90–1.44)	1.20	(0.85–1.69)	1.77	(1.06–2.97)	0.03
Myocardial infarction	1.06	(0.56–2.01)	1	1.18	(0.91–1.53)	1.49	(1.00–2.22)	1.45	(0.73–2.85)	0.02
Angina pectoris	1.16	(0.66–2.04)	1	1.25	(0.99–1.59)	1.57	(1.12–2.20)	2.16	(1.13–4.14)	<0.01
Thrombosis	1.07	(0.57–1.98)	1	1.20	(0.95–1.52)	1.45	(1.01–2.07)	3.24	(1.94–5.41)	<0.01
MI – angina pectoris – thrombosis1	1.11	(0.75–1.63)	1	1.20	(1.03–1.40)	1.49	(1.18–1.87)	2.45	(1.67–3.59)	<0.01

Data from the Italian National Health Survey, ISTAT, 1999–2000.

*Adjusted for age, education level and smoking habits

‡ Reference category

**p for trend were evaluated excluding the underweight group

^1 ^At least one of the indicated diseases

MI, myocardial infarction

Osteoarthritis was the most common disease across all BMI classes in both genders, but especially among women (27.9% vs 16.4% in men), with a striking 50.1% prevalence among severely obese women, almost twice as high as in the corresponding BMI class in men (27.3%).

Hypertension was the second most prevalent disease in both sexes, and clearly more frequent in women than men (16.3% vs 12.4%). Overweight and obese subjects were at significantly increased risk of hypertension in both genders, with a significant positive linear trend (Tables 3 and 4).

The prevalence of diabetes mellitus was also associated with BMI class, with a slightly higher prevalence among women (4.8% vs 4.3%). In both sexes overweight and obese subjects were at significant risk for diabetes compared to normal-weight subjects, with a significant linear trend (Tables 3 and 4); again this was more evident in women.

Myocardial infarction was positively associated with BMI in both sexes, with a higher prevalence in men than in women (1.9% vs 0.9%). The positive linear trend was strongest in men, with an OR of 5.44 for severely obese versus normal-weight subjects; this was the highest risk observed among all the considered diseases (Table 3).

A statistically significant positive trend in both sexes was evident between BMI and prevalence of angina pectoris and varices, although with different figures.

Thrombosis showed a very similar trend in both genders with a more than doubled prevalence in men compared to women. Only obese and severely obese subjects were at significant risk in both sexes.

To account for a overall cardiovascular disease pattern we defined a composed group including subjects having at least one event among myocardial infarction, angina pectoris and thrombosis. Composite cardiovascular diseases were more prevalent among men, especially obese II/III, with a similar and clear positive trend in both genders (Tables 3 and 4).

No strong positive association between the prevalence of cancer and categories of BMI was evident, particularly in men.

**Figure 1:**
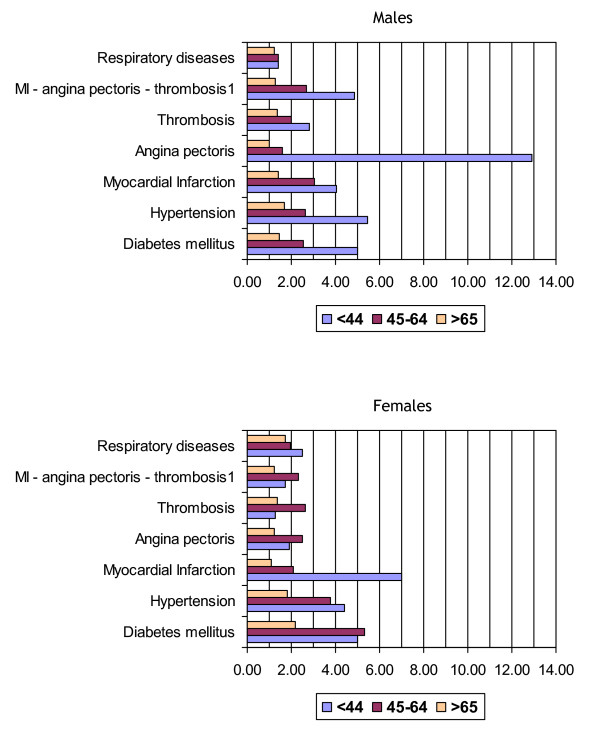
**Sex- and age-specific odds ratio estimates for selected diseases in obese individuals (class I, II and III: BMI ≥30) vs normal-weight individuals.** Estimates were adjusted for education level and smoking habits. Data from the Italian National Health Survey, ISTAT, 1999–2000. 1 At least one of the indicated diseases.

Figure 1 shows OR estimates for obese (I, II and III) individuals of both sexes in three separate age groups for some diseases whose overall prevalence was significantly elevated in the obese groups. In general, for these relevant diseases the ORs tended to be higher in the younger age group and decreased steadily with increasing age.

## Discussion

This study aimed to evaluate the association between overweight and obesity and several chronic diseases within a recent nationally representative survey conducted in Italy. The most recent definition of overweight and obesity was used in our analysis [14,15], with a small adjustment given the very small prevalence of extremely high obesity (class III).

When we compared our data with a recent analysis on a US national survey [9], a clear difference emerged in the overall proportion of overweight or obese subjects, especially among women, between the US population (63% of men and 55% of women) and the Italian population (52.7% of men and 35.3% of women), considering only subjects aged 25 or above for comparability.

Compared with a previous, similar study in the Italian population [21] based on a survey conducted in 1983, the BMI distribution had changed substantially, with a former estimated prevalence of overweight or obesity of 34.1% and 21.3%, respectively, in men and women versus our estimates of 40.6% in men and 24.6% in women (in the 25–29.9-year age group). The underweight and obese class distributions were roughly similar, notwithstanding the different BMI class categorization. Although the estimates in the paper by the Negri and colleagues [21] did not account for population weights, the previous differences suggest an increase in the prevalence of overweight among Italian adults, consistent with the general trend in Western countries, but apparently not in the prevalence of severe obesity (prevalence about 7% in both Italian studies).

Fourteen groups of chronic diseases were examined. Among men, diabetes mellitus, hypertension, myocardial infarction, angina pectoris, thyroid diseases, osteoarthritis, and lower back pain and sciatica were significantly more prevalent in overweight subjects, while all diseases except osteoporosis and cancer had a significantly elevated OR in the obese I and/or obese II/III groups. Considering the linear trend, the prevalence of all diseases, with the exclusion of bronchial asthma, osteoporosis and cancer, showed a significant and positive association with BMI classes. Thyroid diseases showed a less clear trend, with a lower OR in the obese II/III group than in the obese I group.

Overweight women had a higher prevalence of all chronic diseases except myocardial infarction, angina pectoris, thrombosis and cancer, while the obese I and/or obese II/III groups had a significantly higher prevalence of all chronic diseases considered. All diseases showed a clear and significant linear trend with the severity of obesity, excluding myocardial infarction where the prevalence did not increase from obese I to obese II/III.

A stronger association of obesity with health status in women than men was also reported in previous studies [21]. This may be explained as a real greater impact of obesity on health in women, but also as a consequence of differential health status self-reporting among genders [22].

It must be underlined that in this study we did not control for levels of physical activity, which may be both a cause and a consequence of obesity, as well as exerting an independent effect on health conditions.

The present work shows all the limitations of a cross-sectional study. In particular, this study design does not allow to define a causal relationship between obesity and disease. Nevertheless, it is hardly reasonable to construe a reverse relation, i.e., the disease being the cause of obesity. Underweight may be associated with chronic conditions in various ways; for example, weight loss may be the consequence of cancer or thyroid diseases and also, though indirectly, of diabetes. For this reason we excluded the underweight category when testing for linear trends.

Despite these limitations, this study provides an instantaneous picture of the burden of obesity, regardless of the mechanism of action, in a large sample representative of the Italian population in terms of age, sex and area of residence.

Obesity is increasingly common in Western countries and its prevalence is rising steadily, with a great impact on society in terms of public health and health expenditure [23]. To our knowledge no recent study has investigated the burden of obesity and its association with such a wide spectrum of chronic diseases on a population-based sample.

The present study documented an increase in the prevalence of overweight among Italian adults in the last decades and an increased prevalence of several chronic conditions in obese and overweight individuals.

Since participants in self-reported studies tend to overestimate their height and overweight subjects tend to underreport their weight, the true prevalence of overweight and obesity were likely to be underestimated [24-26]. Recent studies found that self-reported information on weight and height underestimated the prevalence of obesity compared with measured data in both sexes. In these studies overestimation of body height and underestimation of body weight tended to increase by increasing age and measured BMI in both sexes [27,28]. This suggests that the estimated positive association between obesity, measured by means of self-reported BMI, and the prevalence of some relevant chronic diseases may be underestimated in this study.

The limitations and uncertainties of some of the collected information will hardly obscure our major finding of a widespread and substantial impact of overweight and obesity on morbidity from several chronic diseases. A general pattern of increasing risk with increasing level of excess weight was observed across genders.

Obesity-related ORs for the most relevant chronic diseases tended to be higher in younger and middle-aged people than in elderly subjects. This suggests that policies aiming to prevent overweight development in young individuals should be promoted, especially because deliberate weight loss in healthy overweight subjects might be hazardous in the long term [29].

According to some authors [8], the global chronic diseases epidemic has a direct cause in obesity, high blood pressure, high blood cholesterol, stress and (as underlying causes) imbalanced diet [30], smoking, excessive alcohol consumption and physical inactivity. This suggests that policies promoting a healthier lifestyle could positively influence obesity rates and therefore lower the chronic disease prevalence.

This kind of policy is becoming more and more important considering the spread of the sedentary lifestyle in Western countries. (For example, the ISTAT National Health Interview Survey [31] estimated that 50.6% of the Italian adult population had a sedentary lifestyle). A policy combining education at school age and community involvement encouraging physical activity, together with a healthy diet, is becoming the principal concern for developed countries in order to reduce the health damaging effects of modern lifestyles [32,33].

## Conclusion

The present study documents an increase in the prevalence of overweight among Italian adults in the last decades and an increased prevalence of several chronic conditions in obese and overweight individuals. A general pattern of positive association between excess weight and chronic diseases was observed for both sexes. To reduce the prevalence of chronic diseases a policy promoting healthier individual lifestyles is becoming more and more desirable.

## Competing interests

The authors declare that they have no competing interests.

## Authors' contributions

SC and MF conceived the work, collected and analyzed the data and helped write the article. AD advised in all stages of the work and helped write the article. All authors read and approved the final manuscript.

## Pre-publication history

The pre-publication history for this paper can be accessed here:


